# In Silico Simulation of Impacts of Metal Nano-Oxides on Cell Viability in THP-1 Cells Based on the Correlation Weights of the Fragments of Molecular Structures and Codes of Experimental Conditions Represented by Means of Quasi-SMILES

**DOI:** 10.3390/ijms24032058

**Published:** 2023-01-20

**Authors:** Alla P. Toropova, Andrey A. Toropov, Natalja Fjodorova

**Affiliations:** 1 Laboratory of Environmental Chemistry and Toxicology, Istituto Di Ricerche Farmacologiche Mario Negri IRCCS, Via Mario Negri, 2, 20156 Milano, Italy; 2 National Institute of Chemistry, SI-1000 Ljubljana, Slovenia

**Keywords:** in silico, Quasi-SMILES, nano-oxides, cell viability in THP-1 cells, Monte Carlo method

## Abstract

A simulation of the effect of metal nano-oxides at various concentrations (25, 50, 100, and 200 milligrams per millilitre) on cell viability in THP-1 cells (%) based on data on the molecular structure of the oxide and its concentration is proposed. We used a simplified molecular input-line entry system (SMILES) to represent the molecular structure. So-called quasi-SMILES extends usual SMILES with special codes for experimental conditions (concentration). The approach based on building up models using quasi-SMILES is self-consistent, i.e., the predictive potential of the model group obtained by random splits into training and validation sets is stable. The Monte Carlo method was used as a basis for building up the above groups of models. The CORAL software was applied to building the Monte Carlo calculations. The average determination coefficient for the five different validation sets was R^2^ = 0.806 ± 0.061.

## 1. Introduction

Nano-safety assessments are often conducted in live organisms, including fish, mice, and rats [1,2]. However, since the European Union and US regulatory authorities consider the development of alternative animal-free testing strategies as the most important challenge for future chemical risk assessment of nano-materials, interest in developing in silico approaches to solving the above task has increased considerably [3]. The lack of structured and systematized databases remains a factor that hinders the development of methods for the simulation of the physicochemical and biochemical behaviour of nano-materials [4,5,6,7,8,9,10]. Nevertheless, work on the creation of methods for assessing nano-safety is being carried out, and their flow is growing [11,12,13,14,15,16,17,18,19,20,21,22]. Nano-safety assessments are in high demand and refer to a wide variety of nano-materials that are increasingly penetrating the everyday life of modern society. One of the main directions of these studies is the development of models of environmental consequences of the use of nano-substances in industry, medicine, and everyday life.

The first attempts to develop in silico approaches to solving the above problem were based on the set of developed molecular descriptors used for traditional substances (organic, inorganic, coordination). At the same time, the combined use of calculated molecular descriptors and experimentally determined numerical data on various physicochemical and biochemical characteristics of nano-materials was used for the development of in silico models of the properties of nano-materials [7].

The development of a special format for presenting data on nano-materials is another concept for building in silico models for nano-materials. This format would be abbreviated ISO-TAB-nano (Investigation/Study/Assay Tabular) [8].

A convenient compromise between the need to have expensive experimental data on nano-materials and the need to quickly evaluate a rapidly expanding list of nano-materials in practical use is the “read-across” approach [9].

Finally, the quasi-SMILES method is an effective method for constructing models of nano-materials’ physicochemical and biochemical behaviour in the absence of systematized databases [23,24,25,26,27,28,29,30,31,32,33,34,35]. The essence of this method in the first approximation is two steps. First, a list of conditions (for example, concentrations of reagents) and circumstances (presence of certain chemical elements) is made, designating each of them with a special code; and secondly, the correlation contribution of each code to some stochastic model of a given endpoint is evaluated using the Monte Carlo method. 

The advantages of using quasi-SMILES are the convenience of formulating problems for in silico modelling and the clarity of the results obtained. The disadvantage of this approach is a significant variance in the results, as a result of which practical reliability can be achieved only when conducting a large number of stochastic computer experiments. It is to be noted that, previously, the index of ideality of correlation and the correlation intensity index have not been used in building models. 

Here, the possibility of using the above-mentioned approach to simulate the impact of nano-oxide metals (in different concentrations) on cell viability in THP-1 cells expressed by a percentage was examined. The calculations described here were carried out with the CORAL software (http://www.insilico.eu/coral, accessed on 10 January 2023, Italy). 

## 2. Results

### 2.1. Models

**Figure 1:**
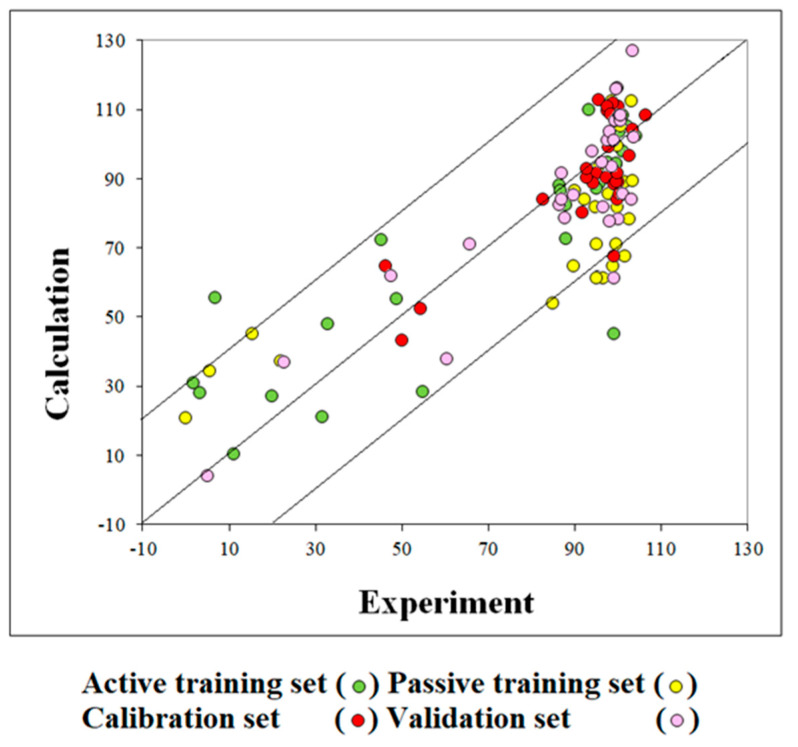
Graphical representation of the model for cell viability in THP-1 cells, which is influenced by different metal nano-oxides under different concentrations.

The computational experiments with five random splits gave models characterized by quite close predictive potential (average determination coefficient R^2^ = 0.806 ± 0.061). Table 1 shows the statistical characteristics of the models. Figure 1 shows the graphical representation of the model for cell viability in THP-1 cells observed for split-1.

### 2.2. Mechanistic Interpretation

Having the numerical data on the correlation weights of codes applied in quasi-SMILES, which was observed in several runs of the Monte Carlo optimization, one is able to detect three categories of these codes:

I. Codes that have a positive value of the correlation weight in all runs. These are promoters of endpoint increase;
II. Codes that have a negative value of the correlation weight in all runs. These are promoters of endpoint decrease;
III. Codes that have both negative and positive values of the correlation weight in different optimization runs. These codes have an unclear role (one cannot classify these features as a promoter of endpoint increase or decrease).

In the case of the analysis of cell viability, promoters of decrease have a practical significance. Table 2 shows the collection of promoters of decrease in cell viability.

### 2.3. Applicability Domain

The applicability domain for the described model calculated with Equation (1) is defined by the so-called statistical defects of quasi-SMILES codes [36]. The percentage of outliers according to the criterion equals 27%, 13%, 17%, 10%, and 13% for split 1, split 2 … split 5, respectively.

## 3. Discussion

In this study, only one additional parameter was available for model development in addition to the molecular structure (transmitted via SMILES), namely the concentration of metal oxide nano-particles. Nevertheless, the results obtained are, in fact, quite reliable models of cell viability in THP-1 cells.

It should be noted that the present approach makes it possible to quite easily improve the predictive potential of the model if additional experimental data are available that can be represented as additional codes for the quasi-SMILES extension. There are examples of works where representative lists of codes for quasi-SMILES are applied in practice [36,37]. Thus, simulation by means of the quasi-SMILES technique claims both simplicity and universality. Consequently, quasi-SMILES can find numerous applications as a tool for developing models for phenomena characterized by an eclectic set of factors influencing them.

It is possible to use the optimal descriptors considered here in conjunction with classical descriptors developed based on information theory ideas, physicochemical parameters (solubility, density, octanol/water distribution coefficient), biochemical characteristics (toxicity, drug effects), or the invariants of the molecular graph (multigraph). The above abilities of the quasi-SMILES technique are especially convenient for a situation related to non-standard objects for the simulation, such as mixtures, peptides, and nano-materials.

No less interesting are the prospects for the development of the objective functions described here used for optimization by the Monte Carlo method. Currently, objective functions based on correlations have been studied, but instead of correlations, the basis for them can be selected entropy values of fuzzy sets generated by various divisions of available data into training and verification subsets.

Like most stochastic approaches, the quasi-SMILES technique makes it possible to analyse existing experimental data, but the possibilities for extrapolating the considered approach are limited. In other words, this approach can be useful only for situations close to those that have been studied in detail in a direct experiment. At the same time, work with experimentally determined data sets can be used for the inverse problem, that is, the selection of experimental characteristics that are promising or, on the contrary, useless, according to the number of available experimental states of the data system under study.

Supplementary materials contain input files for the five splits examined here, together with the CORAL method used in this work.

## 4. Materials and Methods

### 4.1. Data

In [3], data on the impact of nano-oxide nano-particles on cell viability in THP-1 cells was tested at eight dilutions (0, 3.1, 6.2, 13, 25, 50, 100, and 200 μg/mL). Non-zero effects of impact on cell viability in THP-1 cells by the mentioned nano-particles were observed starting from a concentration of just 25. Only non-zero effects were used to build the model. Under such circumstances, the total number of situations (oxide–concentration–cell viability) equals 120. Quasi-SMILES represents each situation. These quasi-SMILES are distributed into four special sub-sets: (i) active training set; (ii) passive training set; (iii) calibration set; and (iv) validation set. Five random splits were examined here as a basis to build up the model of cell viability in THP-1 cells. Each above sub-set contains about 25% of the total list of quasi-SMILES.

Each of the above sets had a defined task. The active training set was used to build the model. Molecular features extracted from quasi-SMILES of the active training set were involved in the process of Monte Carlo optimization aimed to provide correlation weights for the above features, which give maximal target function value, which was calculated using descriptors (the sum of the correlation weights), and endpoint values on the active training set. The task of the passive training set is to check whether the model obtained for the active training set is satisfactory for quasi-SMILES which were not involved in the active training set. The calibration set should detect the start of overtraining (overfitting). The optimization must stop if overtraining starts. After stopping the optimization procedure, the validation set was used to assess the predictive potential of the obtained model.

**Figure 2:**
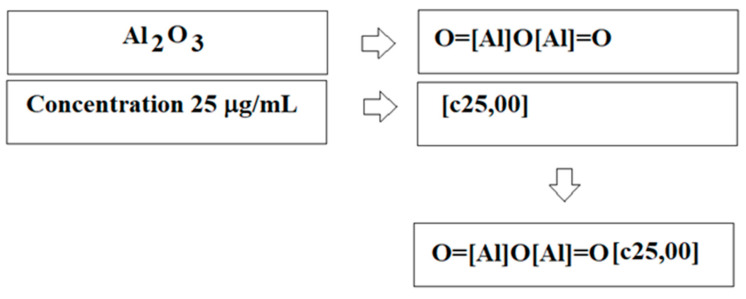
The scheme of building up quasi-SMILES for the situation where the impact of nano-oxide of aluminium in concentration 25 mg/mL is examined.

**Figure 3:**
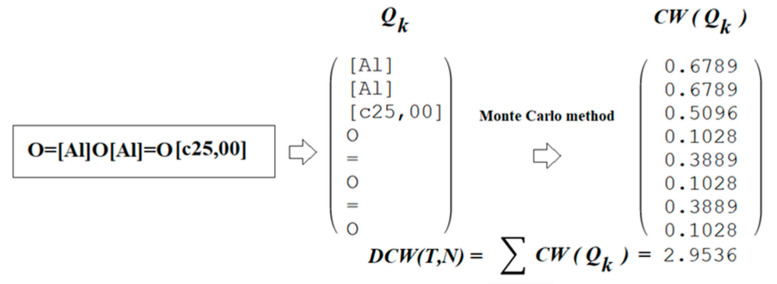
The generalized scheme of calculation of the optimal descriptor based on the correlation weights (*CW*) of codes of quasi-SMILES (i.e., *Q_k_*); the correlation weights *CW*(*Q_k_*) are obtained by the Monte Carlo method.

Figure 2 demonstrates the generalized scheme of construction of quasi-SMILES for the above-mentioned arbitrary situation. Figure 3 includes the general scheme of applying quasi-SMILES (Q_k_) codes to calculate the optimal descriptor for a defined arbitrary situation. 

Table 3 contains split-1 for the total list of quasi-SMILES together with experimental and calculated values of cell viability in THP-1 cells.

### 4.2. Optimal Descriptor

The optimal descriptor is the sum of the correlation weights of the quasi-SMILES codes obtained by the Monte Carlo method (Figure 3). The values of the optimal descriptor serve as the basis for the model of cell viability calculated by the formula

(1) $$cell\;viability_{k}=C_{0}+C_{1}\times DCW\left(T,N\right)$$

The optimal descriptor depends on the style of the Monte Carlo optimization. *T* and *N* are parameters of the optimization procedure. *T* is a threshold applied to define rare codes; if *T* = 1, this means that codes absent in the active training set are rare. The rare codes are not involved in the modelling process (their correlation weights are zero). *N* is the number of epochs in the Monte Carlo optimization. 

### 4.3. Monte Carlo Method

Equation (1) needs the numerical data of the above correlation weights. Monte Carlo optimization is a tool to calculate those correlation weights. Here, two target functions for the Monte Carlo optimization are examined:

(2) $$TF_{0}=r_{AT}+r_{PT}-\left|r_{AT}-r_{PT}\right|\times 0.1$$

(3) $$TF_{1}=TF_{0}+(IIC_{\;}+CII_{\;})\times 0.3$$

The $r_{AT}$ and $r_{PT}$ are correlation coefficients between the observed and predicted endpoints for the active and passive training sets, respectively. The *IIC* is the index of ideality of correlation [33,34]. The *IIC* is calculated using data from the calibration set as follows:

IIC =Rmin(M−AEC,M+AEC) max(M−AEC,M+AEC) minx,y=x, if x<yy,otherwisemaxx,y=x, if x>yy,otherwiseM−AEC=1N−∑∆k, N− is the number of ∆k<0M+AEC=1N+∑∆k, N+ is the number of ∆k≥0Δk=observedk−calculatedk

The *observed_k_* and *calculated_k_* are corresponding values of the endpoint.

The correlation intensity index (*CII*), similar to the above *IIC*, was developed as a tool to improve the quality of the Monte Carlo optimization aimed at building up QSPR/QSAR models. The *CII* is calculated as follows:

$$\begin{array}{l} CII_{C}=1-\sum Protest_{k}\\ Protest_{k}=\left\{\begin{array}{cc} R_{k}^{2}-R^{2}, & if\;R_{k}^{2}-R^{2}>0\\ 0, & otherwise \end{array}\right.\; \end{array}$$

*R*^2^ is the correlation coefficient for a set that contains n substances. $R_{k}^{2}$ is the correlation coefficient for *n* − 1 substances of a set after removal of the *k*-th substance. Hence, if ($R_{k}^{2}-R^{2}$) is larger than zero, the *k*-th substance is an “oppositionist” for the correlation between experimental and predicted values of the set. A small sum of “protests” means a more “intensive” correlation.

The Monte Carlo method aims to minimize the target functions [37], *TF*_1_, based on the application of two new criteria of predictive potential: the index of ideality of correlation [33,34] and correlation intensity index [38,39].

## 5. Conclusions

The quasi-SMILES technique gives quite satisfactory models for cell viability in THP-1 cells, as we have shown the reproducibility of the predictive potential of corresponding models obtained for different splits into sets of training and validation sets. There is variation in the statistical characteristics of the above models; however, this variation is not too large. In other words, the results can be assessed as acceptable for practical use. In addition, that the predictive potential of models can be improved by applying the index of ideality of correlation and the correlation intensity index is confirmed.

## Figures and Tables

**Table 1:** The statistical characteristics of models for cell viability were observed for five random splits.

	*Set* *	*n*	*R* ^2^	*CCC*	*IIC*	*CII*	*Q* ^2^	*RMSE*	*F*
**Split1**	A	29	0.7094	0.8300	0.6843	0.8115	0.6683	19.6	66
**N_CW_ = 25**	P	31	0.6104	0.6880	0.7323	0.7830	0.5186	21.5	45
	C	29	0.5656	0.7312	0.7500	0.7744	0.4437	12.9	35
	V	31	0.7226	-	-	-	-	13.7	
**Split2**	A	32	0.7602	0.8638	0.6782	0.8582	0.7179	17.6	95
**N_CW_ = 28**	P	30	0.6793	0.7287	0.4444	0.8133	0.4913	16.2	59
	C	29	0.5281	0.6999	0.7261	0.8126	0.4225	14.5	30
	V	29	0.8541	-	-	-	-	14.3	
**Split3**	A	29	0.7751	0.8733	0.7153	0.8868	0.7434	18.1	93
**N_CW_ = 27**	P	31	0.6325	0.6949	0.6134	0.7897	0.5575	23.2	50
	C	29	0.5639	0.5557	0.7509	0.8253	0.3264	13.5	35
	V	31	0.7790	-	-	-	-	10.9	
**Split4**	A	31	0.7035	0.8260	0.6907	0.8278	0.6678	21.5	69
**N_CW_ = 27**	P	28	0.7345	0.1563	0.0408	0.8449	0.6879	31.8	72
	C	31	0.6849	0.8205	0.8275	0.8654	0.6012	12.6	63
	V	30	0.7801	-	-	-	-	15.5	
**Split5**	A	29	0.7065	0.8280	0.6829	0.8274	0.6571	18.9	65
**N_CW_ = 28**	P	29	0.8444	0.7829	0.6637	0.9040	0.8239	20.9	146
	C	31	0.6057	0.6661	0.7779	0.8176	0.2765	11.8	45
	V	31	0.8964	-	-	-	-	7.0	

* A = Active training set; P = Passive training set; C = Calibration set; V = Validation set; *n* = the number of quasi-SMILES in a set; *R*^2^ = the determination coefficient; *CCC* = the concordance correlation coefficient; *IIC* = the index of ideality of correlation; *CII* = correlation intensity index; *Q*^2^ = cross-validated leave-one out *R*^2^; *RMSE* = root mean squared error; *F* = Fischer *F*-ratio, N_CW_ = the number of parameters involved in the Monte Carlo optimization.

**Table 2:** Promoters (↓) of decreased cell viability in THP-1 cells, according to computational experiments with five random splits.

	Split1	Split2	Split3	Split4	Split5
**[Mn]**	↓	↓	↓	↓	↓
**[Co]**	↓	↓	↓	↓	↓
**[Cu]**	↓	↓	-	-	-
**[Zn]**	-	-	-	↓	-
**[c200,00]**	-	-	↓	↓	-

**Table 3:** The list of quasi-SMILES, experimental and calculated percentage of cell viability in THP-1 cells. A = active training set; P = passive training set; C = calibration set; V = validation set.

Set	ID	Quasi-SMILES	Experiment (%)	Calculation (%)
C	1	O=[Al]O[Al]=O[c25,00]	102.7800	134.3224
V	2	O=[Al]O[Al]=O[c50,00]	103.4400	126.9137
V	3	O=[Al]O[Al]=O[c100,00]	99.8800	116.2402
A	4	O=[Al]O[Al]=O[c200,00]	93.2600	109.9123
P	5	O=[Bi]O[Bi]=O[c25,00]	98.6300	112.2648
A	6	O=[Bi]O[Bi]=O[c50,00]	100.7300	104.8562
A	7	O=[Bi]O[Bi]=O[c100,00]	99.6300	94.1827
A	8	O=[Bi]O[Bi]=O[c200,00]	100.2600	87.8548
P	9	O=[Ge]=O[c25,00]	97.8300	85.6033
P	10	O=[Ge]=O[c50,00]	100.1900	78.1946
P	11	O=[Ge]=O[c100,00]	99.5000	67.5211
P	12	O=[Ge]=O[c200,00]	96.7000	61.1932
C	13	[Co]=O[c25,00]	54.4100	52.4457
P	14	[Co]=O[c50,00]	15.5500	45.0370
P	15	[Co]=O[c100,00]	5.6600	34.3635
A	16	[Co]=O[c200,00]	3.2600	28.0356
A	17	[Co]=O.O=[Co]O[Co]=O[c25,00]	95.4400	61.3872
P	18	[Co]=O.O=[Co]O[Co]=O[c50,00]	84.9300	53.9786
C	19	[Co]=O.O=[Co]O[Co]=O[c100,00]	49.9600	43.3051
V	20	[Co]=O.O=[Co]O[Co]=O[c200,00]	22.6500	36.9772
P	21	O=[Cr]O[Cr]=O[c25,00]	101.7700	89.0326
P	22	O=[Cr]O[Cr]=O[c50,00]	94.8500	81.6240
V	23	O=[Cr]O[Cr]=O[c100,00]	65.8100	70.9505
C	24	O=[Cr]O[Cr]=O[c200,00]	46.3600	64.6226
A	25	[Cu]=O[c25,00]	99.1700	45.0965
V	26	[Cu]=O[c50,00]	60.4100	37.6879
A	27	[Cu]=O[c100,00]	19.8700	27.0144
P	28	[Cu]=O[c200,00]	0.1000	20.6865
C	29	O=[Dy]O[Dy]=O[c25,00]	97.6000	109.6235
A	30	O=[Dy]O[Dy]=O[c50,00]	104.1500	102.2148
C	31	O=[Dy]O[Dy]=O[c100,00]	95.0600	91.5413
V	32	O=[Dy]O[Dy]=O[c200,00]	89.7000	85.2134
C	33	O=[Er]O[Er]=O[c25,00]	100.1600	89.0326
V	34	O=[Er]O[Er]=O[c50,00]	96.5800	81.6240
P	35	O=[Er]O[Er]=O[c100,00]	95.1000	70.9505
P	36	O=[Er]O[Er]=O[c200,00]	89.7400	64.6226
V	37	O=[Eu]O[Eu]=O[c25,00]	99.4800	106.8651
P	38	O=[Eu]O[Eu]=O[c50,00]	99.9800	99.4564
A	39	O=[Eu]O[Eu]=O[c100,00]	95.7800	88.7829
V	40	O=[Eu]O[Eu]=O[c200,00]	86.5300	82.4550
C	41	[Fe+3].[Fe+3].[O-2].[O-2].[O-2][c25,00]	99.9200	108.3871
C	42	[Fe+3].[Fe+3].[O-2].[O-2].[O-2][c50,00]	98.8800	100.9784
C	43	[Fe+3].[Fe+3].[O-2].[O-2].[O-2][c100,00]	97.3700	90.3049
C	44	[Fe+3].[Fe+3].[O-2].[O-2].[O-2][c200,00]	99.9200	83.9770
C	45	[Fe]=O.O=[Fe]O[Fe]=O[c25,00]	95.6700	112.7077
P	46	[Fe]=O.O=[Fe]O[Fe]=O[c50,00]	100.6200	105.2991
A	47	[Fe]=O.O=[Fe]O[Fe]=O[c100,00]	97.5800	94.6256
C	48	[Fe]=O.O=[Fe]O[Fe]=O[c200,00]	99.0300	88.2977
V	49	[Gd+3].[Gd+3].[O-2].[O-2].[O-2][c25,00]	100.3700	108.3871
V	50	[Gd+3].[Gd+3].[O-2].[O-2].[O-2][c50,00]	98.1200	100.9784
P	51	[Gd+3].[Gd+3].[O-2].[O-2].[O-2][c100,00]	94.3400	90.3049
V	52	[Gd+3].[Gd+3].[O-2].[O-2].[O-2][c200,00]	86.9100	83.9770
C	53	O=[Hf]=O[c25,00]	100.2900	85.6033
P	54	O=[Hf]=O[c50,00]	102.6100	78.1946
P	55	O=[Hf]=O[c100,00]	101.7900	67.5211
P	56	O=[Hf]=O[c200,00]	95.0000	61.1932
V	57	[In+3].[In+3].[O-2].[O-2].[O-2][c25,00]	100.6200	106.6455
C	58	[In+3].[In+3].[O-2].[O-2].[O-2][c50,00]	97.9200	99.2368
C	59	[In+3].[In+3].[O-2].[O-2].[O-2][c100,00]	94.2200	88.5633
A	60	[In+3].[In+3].[O-2].[O-2].[O-2][c200,00]	87.9600	82.2354
V	61	[La+3].[La+3].[O-2].[O-2].[O-2][c25,00]	100.7500	108.3871
V	62	[La+3].[La+3].[O-2].[O-2].[O-2][c50,00]	97.5400	100.9784
C	63	[La+3].[La+3].[O-2].[O-2].[O-2][c100,00]	92.7000	90.3049
C	64	[La+3].[La+3].[O-2].[O-2].[O-2][c200,00]	82.8000	83.9770
A	65	O=[Mn]=O[c25,00]	48.8900	55.2509
A	66	O=[Mn]=O[c50,00]	32.7700	47.8423
P	67	O=[Mn]=O[c100,00]	22.0400	37.1688
A	68	O=[Mn]=O[c200,00]	1.7500	30.8409
A	69	O=[Mn]O[Mn]=O[c25,00]	54.9500	28.3280
A	70	O=[Mn]O[Mn]=O[c50,00]	31.5800	20.9193
A	71	O=[Mn]O[Mn]=O[c100,00]	11.1200	10.2458
V	72	O=[Mn]O[Mn]=O[c200,00]	5.1400	3.9179
C	73	O=[Nd]O[Nd]=O[c25,00]	100.2400	110.9428
A	74	O=[Nd]O[Nd]=O[c50,00]	100.3200	103.5342
P	75	O=[Nd]O[Nd]=O[c100,00]	95.3200	92.8607
P	76	O=[Nd]O[Nd]=O[c200,00]	89.9300	86.5328
P	77	[O-2].[Ni+2][c25,00]	103.3200	112.4964
A	78	[O-2].[Ni+2][c50,00]	102.3000	105.0877
A	79	[O-2].[Ni+2][c100,00]	99.7700	94.4142
A	80	[O-2].[Ni+2][c200,00]	86.6000	88.0863
C	81	[Ni+3].[Ni+3].[O-2].[O-2].[O-2][c25,00]	102.7800	96.5984
P	82	[Ni+3].[Ni+3].[O-2].[O-2].[O-2][c50,00]	103.4400	89.1897
V	83	[Ni+3].[Ni+3].[O-2].[O-2].[O-2][c100,00]	87.7500	78.5162
A	84	[Ni+3].[Ni+3].[O-2].[O-2].[O-2][c200,00]	45.3300	72.1883
C	85	O=[Sb]O[Sb]=O[c25,00]	99.7200	89.0326
P	86	O=[Sb]O[Sb]=O[c50,00]	99.9100	81.6240
P	87	O=[Sb]O[Sb]=O[c100,00]	99.6800	70.9505
P	88	O=[Sb]O[Sb]=O[c200,00]	98.8300	64.6226
V	89	O=[Sm]O[Sm]=O[c25,00]	99.6700	115.8481
A	90	O=[Sm]O[Sm]=O[c50,00]	101.1200	108.4395
V	91	O=[Sm]O[Sm]=O[c100,00]	94.0300	97.7660
V	92	O=[Sm]O[Sm]=O[c200,00]	86.9700	91.4381
C	93	O=[Sn]=O[c25,00]	98.8000	111.6224
C	94	O=[Sn]=O[c50,00]	103.5400	104.2137
V	95	O=[Sn]=O[c100,00]	98.7200	93.5402
A	96	O=[Sn]=O[c200,00]	95.1500	87.2123
V	97	O=[Ti]=O[c25,00]	101.2200	85.6033
V	98	O=[Ti]=O[c50,00]	100.2700	78.1946
C	99	O=[Ti]=O[c100,00]	99.2700	67.5211
V	100	O=[Ti]=O[c200,00]	99.2300	61.1932
V	101	O=[W](=O)=O[c25,00]	103.8200	102.0069
V	102	O=[W](=O)=O[c50,00]	96.3200	94.5982
V	103	O=[W](=O)=O[c100,00]	103.3000	83.9248
V	104	O=[W](=O)=O[c200,00]	98.2600	77.5969
C	105	O=[Y]O[Y]=O[c25,00]	97.7000	110.9296
V	106	O=[Y]O[Y]=O[c50,00]	98.1200	103.5209
C	107	O=[Y]O[Y]=O[c100,00]	92.8300	92.8474
A	108	O=[Y]O[Y]=O[c200,00]	86.7300	86.5195
C	109	[O-2].[O-2].[O-2].[Yb+3].[Yb+3][c25,00]	106.5900	108.3871
V	110	[O-2].[O-2].[O-2].[Yb+3].[Yb+3][c50,00]	99.1900	100.9784
P	111	[O-2].[O-2].[O-2].[Yb+3].[Yb+3][c100,00]	99.4400	90.3049
P	112	[O-2].[O-2].[O-2].[Yb+3].[Yb+3][c200,00]	92.3800	83.9770
P	113	[Zn]=O[c25,00]	91.8300	80.0461
A	114	[Zn]=O[c50,00]	87.9600	72.6374
V	115	[Zn]=O[c100,00]	47.6400	61.9639
A	116	[Zn]=O[c200,00]	6.7600	55.6360
C	117	O=[Zr]=O[c25,00]	99.6500	115.9612
C	118	O=[Zr]=O[c50,00]	98.4900	108.5525
A	119	O=[Zr]=O[c100,00]	101.0700	97.8790
P	120	O=[Zr]=O[c200,00]	100.0200	91.5511

## Data Availability

Technical details on the five models are available in the Supplementary materials section.

## Supplementary Materials

The following supporting information can be downloaded at: https://www.mdpi.com/article/10.3390/ijms24032058/s1.

## References

[1] Toropova A.P., Toropov A.A. (2017). Nano-QSAR in cell biology: Model of cell viability as a mathematical function of available eclectic data. J. Theor. Biol..

[2] Li J., Wang C., Yue L., Chen F., Cao X., Wang Z. (2022). Nano-QSAR modeling for predicting the cytotoxicity of metallic and metal oxide nanoparticles: A review. Ecotoxicol. Environ. Saf..

[3] Huang Y., Li X., Cao J., Wei X., Li Y., Wang Z., Cai X., Li R., Chen J. (2022). Use of dissociation degree in lysosomes to predict metal oxide nanoparticle toxicity in immune cells: Machine learning boosts nano-safety assessment. Environ. Int..

[4] Yan X., Sedykh A., Wang W., Yan B., Zhu H. (2020). Construction of a web-based nanomaterial database by big data curation and modeling friendly nanostructure annotations. Nat. Commun..

[5] Mills K.C., Murry D., Guzan K.A., Ostraat M.L. (2014). Nanomaterial registry: Database that captures the minimal information about nanomaterial physico-chemical characteristics. J. Nanoparticle Res..

[6] Panneerselvam S., Choi S. (2014). Nanoinformatics: Emerging databases and available tools. Int. J. Mol. Sci..

[7] Fourches D., Pu D., Tassa C., Weissleder R., Shaw S.Y., Mumper R.J., Tropsha A. (2010). Quantitative nanostructure-Activity relationship modeling. ACS Nano..

[8] Thomas D.G., Gaheen S., Harper S.L., Fritts M., Klaessig F., Hahn-Dantona E., Paik D., Pan S., Stafford G.A., Freund E.T. (2013). ISA-TAB-Nano: A Specification for Sharing Nanomaterial Research Data in Spreadsheet-based Format. BMC Biotechnol..

[9] Gajewicz A., Jagiello K., Cronin M.T.D., Leszczynski J., Puzyn T. (2017). Addressing a bottle neck for regulation of nanomaterials: Quantitative read-across (Nano-QRA) algorithm for cases when only limited data is available. Environ. Sci. Nano.

[10] Krug H.F. (2022). Collection of Controlled Nanosafety Data—The CoCoN-Database, a Tool to Assess Nanomaterial Hazard. Nanomaterials.

[11] Jimenez-Cruz C.A., Kang S.-g., Zhou R. (2014). Large scale molecular simulations of nanotoxicity. WIREs Syst. Biol. Med..

[12] Toropova A.P., Toropov A.A., Veselinović A.M., Veselinović J.B., Benfenati E., Leszczynska D., Leszczynski J. (2016). Nano-QSAR: Model of mutagenicity of fullerene as a mathematical function of different conditions. Ecotoxicol. Environ. Saf..

[13] Piane M.D., Potthoff S., Brinker C.J., Colombi Ciacchi L. (2018). Molecular Dynamics Simulations of the Silica-Cell Membrane Interaction: Insights on Biomineralization and Nanotoxicity. J. Phys. Chem. C.

[14] Buglak A.A., Zherdev A.V., Dzantiev B.B. (2019). Nano-(Q)SAR for Cytotoxicity Prediction of Engineered Nanomaterials. Molecules.

[15] González-Durruthy M., Giri A.K., Moreira I., Concu R., Melo A., Ruso J.M., Cordeiro M.N.D.S. (2020). Computational modeling on mitochondrial channel nanotoxicity. Nano Today.

[16] Toropova A.P., Toropov A.A. (2020). Fullerenes C60 and C70: A model for solubility by applying the correlation intensity index. Fuller. Nanotub. Carbon Nanostruct..

[17] Wu Y.-H., Ho S.-Y., Wang B.-J., Wang Y.-J. (2020). The recent progress in nanotoxicology and nanosafety from the point of view of both toxicology and ecotoxicology. Int. J. Mol. Sci..

[18] Mukhopadhyay T.K., Ghosh A., Datta A. (2021). Molecular Dynamics Simulations Reveal Orientation-Dependent Nanotoxicity of Black Phosphorene toward Dimeric Proteins. ACS Appl. Nano Mater..

[19] Huang H.-J., Lee Y.-H., Hsu Y.-H., Liao C.-T., Lin Y.-F., Chiu H.-W. (2021). Current strategies in assessment of nanotoxicity: Alternatives to in vivo animal testing. Int. J. Mol. Sci..

[20] Tsukanov A.A., Turk B., Vasiljeva O., Psakhie S.G. (2022). Computational Indicator Approach for Assessment of Nanotoxicity of Two-Dimensional Nanomaterials. Nanomaterials.

[21] Thwala M.M., Afantitis A., Papadiamantis A.G., Tsoumanis A., Melagraki G., Dlamini L.N., Ouma C.N.M., Ramasami P., Harris R., Puzyn T. (2022). Using the Isalos platform to develop a (Q)SAR model that predicts metal oxide toxicity utilizing facet-based electronic, image analysis-based, and periodic table derived properties as descriptors. Struct. Chem..

[22] Fjodorova N., Novič M., Venko K., Drgan V., Rasulev B., Türker Saçan M., Sağ Erdem S., Tugcu G., Toropova A.P., Toropov A.A. (2022). How fullerene derivatives (FDs) act on therapeutically important targets associated with diabetic diseases. Comput. Struct. Biotechnol. J..

[23] Ahmadi S., Aghabeygi S., Farahmandjou M., Azimi N. (2021). The predictive model for band gap prediction of metal oxide nanoparticles based on quasi-SMILES. Struct. Chem..

[24] Ahmadi S. (2020). Mathematical modeling of cytotoxicity of metal oxide nanoparticles using the index of ideality correlation criteria. Chemosphere.

[25] Trinh T.X., Choi J.-S., Jeon H., Byun H.-G., Yoon T.-H., Kim J. (2018). Quasi-SMILES-Based Nano-Quantitative Structure-Activity Relationship Model to Predict the Cytotoxicity of Multiwalled Carbon Nanotubes to Human Lung Cells. Chem. Res. Toxicol..

[26] Choi J.-S., Ha M.K., Trinh T.X., Yoon T.-H., Byun H.-G. (2018). Towards a generalized toxicity prediction model for oxide nanomaterials using integrated data from different sources. Sci. Rep..

[27] Choi J.-S., Trinh T.X., Yoon T.-H., Kim J., Byun H.-G. (2019). Quasi-QSAR for predicting the cell viability of human lung and skin cells exposed to different metal oxide nanomaterials. Chemosphere.

[28] Toropova A.P., Toropov A.A. (2013). Optimal descriptor as a translator of eclectic information into the prediction of membrane damage by means of various TiO_2_ nanoparticles. Chemosphere.

[29] Toropov A.A., Toropova A.P. (2014). Optimal descriptor as a translator of eclectic data into endpoint prediction: Mutagenicity of fullerene as a mathematical function of conditions. Chemosphere.

[30] Jafari K., Fatemi M.H. (2020). A new approach to model isobaric heat capacity and density of some nitride-based nanofluids using Monte Carlo method. Adv. Powder Technol..

[31] Jafari K., Fatemi M.H. (2020). Application of nano-quantitative structure–property relationship paradigm to develop predictive models for thermal conductivity of metal oxide-based ethylene glycol nanofluids. J. Therm. Anal. Calorim..

[32] Manganelli S., Benfenati E., Gilbert D., Friedrich O. (2017). Nano-QSAR model for predicting cell viability of human embryonic kidney cells. Cell Viability Assays. Methods in Molecular Biology.

[33] Toropova A.P., Toropov A.A. (2019). Does the Index of Ideality of Correlation Detect the Better Model Correctly?. Mol. Inform..

[34] Toropova A.P., Toropov A.A. (2019). QSPR and nano-QSPR: What is the difference?. J. Mol. Struct..

[35] Toropova A.P., Toropov A.A., Benfenati E. (2019). QSPR as a random event: Solubility of fullerenes C [60] and C [70]. Fuller. Nanotub. Carbon Nanostruct..

[36] Toropov A.A., Kjeldsen F., Toropova A.P. (2022). Use of quasi-SMILES to build models based on quantitative results from experiments with nanomaterials. Chemosphere.

[37] Toropova A.P., Toropov A.A., Fjodorova N. (2022). Quasi-SMILES for predicting toxicity of Nano-mixtures to *Daphnia Magna*. NanoImpact.

[38] Toropov A.A., Toropova A.P. (2020). Correlation intensity index: Building up models for mutagenicity of silver nanoparticles. Sci. Total Environ..

[39] Selvestrel G., Lavado G.J., Toropova A.P., Toropov A.A., Gadaleta D., Marzo M., Baderna D., Benfenati E. (2022). Monte Carlo Models for Sub-Chronic Repeated-Dose Toxicity: Systemic and Organ-Specific Toxicity. Int. J. Mol. Sci..

